# Impact of Radioactive Iodine Treatment on Long-Term Relative Survival in Patients with Papillary and Follicular Thyroid Cancer: A SEER-Based Study Covering Histologic Subtypes and Recurrence Risk Categories

**DOI:** 10.2967/jnumed.124.269091

**Published:** 2025-04

**Authors:** Henning Weis, Jasmin Weindler, Katharina Schmidt, Martin Hellmich, Alexander Drzezga, Matthias Schmidt

**Affiliations:** 1 Department of Nuclear Medicine, Faculty of Medicine, University Hospital of Cologne, University of Cologne, Cologne, Germany;; 2 German Aerospace Center, Institute of Aerospace Medicine, Cologne, Germany; and; 3 Institute of Medical Statistics and Computational Biology, Faculty of Medicine, University Hospital of Cologne, University of Cologne, Cologne, Germany

**Keywords:** papillary thyroid cancer, follicular thyroid cancer, radioiodine treatment, relative survival, risk stratification

## Abstract

For patients with differentiated thyroid cancer (DTC), that is, papillary thyroid cancer (PTC) and follicular thyroid cancer (FTC), the American Thyroid Association and European Thyroid Association generally recommend radioactive iodine (RAI) therapy after surgery only for high-risk patients. For intermediate-risk patients, RAI therapy is recommended only as a should-be-considered option. For low-risk patients, RAI therapy is not routinely recommended. Other countries, such as Germany, are more in favor of using RAI. Thus, RAI therapy remains a matter of controversial debate, because prospective long-term data on survival are scarce. **Methods:** We retrospectively compared long-term relative survival in DTC cohorts treated with and without RAI. From the Surveillance, Epidemiology, and End Results Program database, 101,087 patients harboring DTC were identified between 2000 and 2020. Patient cohorts were subdivided based on histology (classical PTC, aggressive variants of PTC, FTC, and minimally invasive FTC). These cohorts were stratified into the following categories: very low risk, low risk, intermediate risk, and high risk. Relative survival was determined for each subgroup. Statistics included a *z*-test specifically developed for comparison of relative survival, testing the long-term effect of RAI therapy (3, 5, and 10 y). **Results:** The relative survival rate is higher or tends to be higher in most subgroups undergoing RAI therapy than in subgroups not undergoing RAI therapy. Even for low-risk minimally invasive FTC, the 10-y relative survival rate tends to be higher by 2.0% (*P* = 0.055). For larger tumor size or lymph node involvement in classical PTC, a 10-y relative survival benefit of 1.3%–2.0% (*P* = 0.045) in the RAI subgroup prevails. In high-risk DTC, benefits in relative survival of up to 30.9% (*P* < 0.05) were observed. Relative survival is not negatively affected in any RAI subgroup. **Conclusion:** In patients with DTC, depending on histology subtype, a benefit in relative survival prevails in low-, intermediate-, and high-risk subgroups that underwent RAI therapy compared with patients who did not undergo RAI therapy. Even in low-risk minimally invasive FTC, a clear trend toward higher survival rates is observed. For PTC, a survival benefit prevails in the presence of lymph node involvement, larger tumor size, or distant metastasis.

## Visual Abstract

**Figure:**
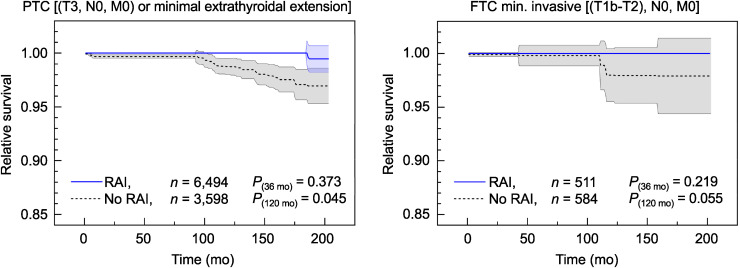

The American Thyroid Association proposed a risk stratification system for disease recurrence for patients with differentiated thyroid cancer (DTC), that is, papillary thyroid cancer (PTC) and follicular thyroid cancer (FTC), who show no structurally identifiable disease after initial therapy. Risk of recurrence is estimated on the basis of TNM classification of malignant tumors, histology, and individual risk factors using distinct risk categories (1). With stronger emphasis on TNM, a similar risk stratification was proposed by the European Thyroid Association (2). An upcoming German guideline will contain a similar risk stratification system but will additionally distinguish the categories of low and very low risk (3). Size and number of lymph nodes are acknowledged as well.

The American Thyroid Association and European Thyroid Association generally recommend radioactive iodine (RAI) therapy after surgery only for high-risk patients, whereas for intermediate-risk patients, RAI therapy is recommended as a should-be-considered treatment option. For very-low-risk and low-risk patients, RAI therapy is not routinely recommended (1,4). However, the usefulness of an adjuvant RAI therapy remains a matter of controversial debate even for low-risk subgroups (3,5,6), because long-term survival data are scarce with partially conflicting results.

Two recent metaanalysis consistently revealed mixed results (7,8), with a trend toward a survival benefit after RAI therapy in overall survival for DTC tumors with a diameter of more than 1 cm (9). If additional risk factors for PTC tumors with a diameter of less than 1 cm prevail (e.g., lymph node involvement), a recent metaanalysis reported a beneficial effect of RAI (10). Another metaanalysis reported that the recurrence rate in intermediate-risk patients receiving successful thyroid ablation with RAI was very low (2%) and was significantly higher without successful thyroid ablation (11). Successful thyroid ablation may be achieved only using RAI, and the authors pointed to the importance of an ongoing risk assessment.

Moreover, recent retrospective studies based on large cancer databases, such as the Surveillance, Epidemiology, and End Results Program (SEER) database, showed a trend toward slightly higher long-term overall survival rates after RAI therapy even in low-risk patients. Yet inconsistencies with regard to cancer-specific survival exist (12–15). No overall survival benefit after RAI therapy was reported for aggressive variants of PTC with a tumor diameter of less than 2 cm based on a national cancer database review (16). Patient subgroups were not stratified to predefined recurrence risk categories in either study.

With regard to the methodology of survival analysis based on population statistics and cancer databases, recent retrospective studies advise cautious interpretation (17): the SEER cancer database allows the calculation of observed all-cause survival (overall survival) and net cancer survival. Net survival estimates the probability of surviving a specific cancer in the absence of other causes of death. Two fundamentally different approaches may be used to calculate net survival: cancer-specific survival relies on the cause-of-death information provided, whereas relative survival uses expected survival tables and measures the death rate in proportion to the expected survivors in a comparable set of cancer-free individuals. Both are valid methodologies yet biased by specific errors. Relative survival is impaired when no suitable life table of cancer-free individual exists. Cancer-specific survival is subject to errors mostly when misclassification of cause of death occurs, occurrences of which, in turn, tend to be higher in rarer cancer sites or older patients. Therefore, Makkar et al. (17) reported that for older patients and potentially for rarer cancer sites, relative survival was considered the preferred survival measure. Here, a rare cancer type (DTC) is investigated, which because of its comparable slow progress tends to cause mortality in an older population. Therefore, we believe relative survival to be the appropriate net survival measure for DTC and proceed to compare relative survival rates.

In addition to retrospective data, only limited prospective data exist. A single prospective study showed that treatment avoiding RAI after surgery was noninferior to a treatment involving RAI for selected low-risk DTC patients. However, the follow-up time was short (3 y), and the histology subgroups consisted of predominantly PTC, with FTC comprising only 3.1% of patients (*n* = 24), rendering long-term prognostication and generalization with regard to histology subtype difficult (18,19).

In general, prospective studies covering the variety of risk categories and histologic subtypes of DTC within the American Thyroid Association and European Thyroid Association risk stratification systems, in particular on a long-term follow-up timescale, hardly seem achievable. In the following, we analyze long-term survival data in patients with DTC (PTC, FTC, and subtypes) with and without RAI therapy based on the large SEER database.

We stratified patients according to recurrence risk categories and histologic subtypes based on current guidelines (3). Our approach, unlike previous database studies, included evaluation of relative survival as a robust measure of response to therapy. Moreover, direct comparisons across subgroups were performed. A *z*-test developed especially to compare relative survival rates (20) was applied to elucidate the potential benefit of RAI for each subgroup.

## MATERIALS AND METHODS

This retrospective cohort analysis included data from the SEER database (SEER Research Plus data, 17 registries, November 2022) between 2000 and 2020. We identified 187,915 patients (age, >20 y) harboring thyroid cancer (primary site code, C73.9 thyroid gland). We further selected patients according to histology (classic PTC, aggressive PTC, and FTC, including minimally invasive and angioinvasive FTC). Details with regard to selection are given in the supplemental materials (supplemental materials are available at http://jnm.snmjournals.org).

On the basis of the ideas in current guidelines (3), each histologic subtype was classified as very low risk (pT1aN0, with pT1a < 1 cm), low risk (pT1bN0M0–pT2N0M0), intermediate risk (pT3N0M0 or minimal extrathyroidal extension, alternatively pT1N1M0–pT3N1M0), or high risk (pT3N1–pT4N1, or M1, or gross extrathyroidal extension). Survival relative to the proportion of expected survivors in a comparable set of cancer-free individuals (relative survival), overall survival, and cancer-specific survival, including its SE, were generated using SEER*Stat software (version 8.4.3; National Cancer Institute) with the default actuarial method (17,21). We generated discrete time intervals of 1 mo up to a maximum follow-up time of 220 mo. Relative survival was estimated using the SEER*Stat default Ederer II method, as used in Makkar et al. (17) and Skyrud et al. (21). (Details with regard to the survival estimate are given in the supplemental materials.)

We included only cases with confirmed malignancy and matching to the SEER expected survival table, identical for overall, cancer-specific, and relative survival (details are given in the supplemental materials). If data on race, age, sex, or date at the time of coding were missing, no matching with a cancer-free subgroup could be performed. As a result, 101,087 patients remained. Nevertheless, a sufficiently large number of patients (*n* > 50) was acquired within most subgroups for adequate analysis (Table 1 and Supplemental Table 1 give the exact numbers initially and followed for more than 10 y). For angioinvasive FTC, a reasonable analysis could not be performed (details are given in the supplemental materials).

**TABLE 1.** RS Differences With and Without RAI Therapy for Studied Cancer Types as Stratified by Recurrence Risk Categories

		Overall (*n*)								Follow-up > 10 y (*n*)				
TNM stage	Cancer type	RAI	No RAI	RAI?to?no RAI absolute difference (%) at 3 y	*z*	*P*	RAI–to–no RAI absolute difference (%) at 5 y	*z*	*P*	RAI	No RAI	RAI–to–no RAI absolute difference (%) at 10 y	*z*	*P*
T1bN0M0–T2N0M0	PTC	12,341	14,773	0.09	3.165	0.002	0.09	2.186	0.029	3,700	2,202	0.09	1.280	0.201
	PTC aggr.	145	117	0.43	0.928	0.353	0.43	0.552	0.581	31	13	−0.37	0.112	0.911
	FTC	979	888	0.73	2.696	0.007	0.73	1.765	0.078	425	318	0.73	1.677	0.094
	FTC min. inv.	511	584	0.10	1.229	0.219	0.19	1.176	0.240	220	130	2.02	1.916	0.055
T3N0M0 or minimal ETE	PTC	6,494	3,598	0.32	−0.891	0.373	0.32	−0.571	0.568	2,461	1,164	1.25	2.009	0.045
	PTC aggr.	178	74	0.37	−0.248	0.804	0.37	−0.173	0.863	72	25	0.29	−0.027	0.978
	FTC	770	528	1.09	0.226	0.821	0.64	0.184	0.854	285	167	3.68	1.547	0.122
	FTC min. inv.	338	291	0.30	0.206	0.836	0.83	1.128	0.259	134	78	1.21	0.036	0.971
T1N1M0–T3N1M0	PTC	17,627	6,241	1.10	3.776	0.000	1.57	2.948	0.003	5,655	1,700	1.95	2.184	0.029
	PTC aggr.	495	137	2.78	1.889	0.059	2.36	1.154	0.248	128	40	1.99	0.708	0.479
	FTC	64	25	17.38	2.673	0.008	12.68	1.635	0.102	17	9	16.45	1.858	0.063
	FTC min. inv.	11	6	0.00	0.000	1.000	0.00	0.000	1.000	3	3	0.00	0.000	1.000
T3N1–T4N1, or M1, or gross ETE	PTC	9,449	3,187	10.26	24.026	0.000	11.25	22.720	0.000	3,197	903	11.89	19.720	0.000
	PTC aggr.	445	131	17.29	5.564	0.000	18.90	5.209	0.000	130	37	16.89	4.278	0.000
	FTC	220	173	34.06	7.874	0.000	39.61	8.148	0.000	44	18	30.91	7.191	0.000
	FTC min. inv.	22	9	10.71	1.554	0.120	8.25	1.030	0.303	5	1	−22.08	0.202	0.840

RS = relative survival; ETE = extrathyroidal extension; PTC aggr. = aggressive variants of PTC; FTC min. inv. = minimally invasive FTC.

Differences in RS were tested using *z*-test particularly suited for comparing RS.

Differences between patients receiving RAI and patients not receiving RAI can visually be assessed pointwise for each subgroup in each survival measure because error bands are included. In addition, the effect of RAI therapy was compared using a SEER default *z*-test developed especially for comparison of relative survival (20) over a predefined period and covering the follow-up intervals of 0–36, 0–60, and 0–120 mo.

The ethics committee of the University of Cologne approved this retrospective study. The requirement to obtain informed consent was waived.

## RESULTS

### Net Survival Measure: Relative Survival

On the basis of the ideas presented in the introduction, we consider relative survival to be an appropriate net survival measure for DTC. We additionally present cancer-specific survival and overall survival for comparability reasons for all investigated histologic subtypes (Supplemental Figs. 1–4). Differences between cancer-specific survival and relative survival appear as expected, with relative survival generally indicating a stronger survival benefit in subgroups that underwent RAI therapy than in no-RAI subgroups (e.g., the intermediate-risk subgroup in Supplemental Fig. 1 and the high-risk subgroup in Supplemental Fig. 3). DTC is a rare cancer so we attribute these observed differences to misclassification of cause of death, which corresponds well to the experience gained in our institution.

### PTC

**FIGURE 1.**
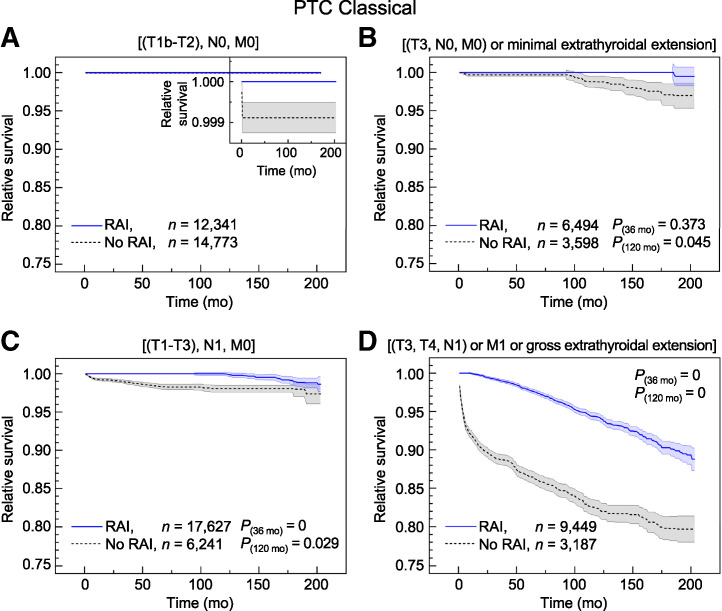
Relative survival including SE band in patients with classical PTC with or without RAI therapy based on SEER cancer database for low-risk (A), intermediate-risk (B and C), and high-risk (D) subgroups. SE band for RAI group in B is smaller than line width below ∼180 mo. Inset in A upscales relative survival measure by order of magnitude, demonstrating at most minor and clinically insignificant differences detected. Therefore, in cases of small tumor size and no lymph node involvement (low-risk subgroups in A), no clinically relevant difference in relative survival prevails. However, with lymph node involvement and larger tumor size (intermediate-risk categories in C and B, respectively), relevant higher long-term survival rates in RAI groups are detected (within a percentage range) and even more so in cases with high risk of recurrence (subgroups in D).

In Figure 1, relative survival rates in classical variants of PTC with and without RAI are depicted for the low-, intermediate-, and high-risk category patients. In the supplemental materials, relative survival for PTC in the very-low-risk category (Supplemental Fig. 1) and relative survival for aggressive variants across risk categories (Supplemental Fig. 2) are depicted. In Table 1 and Supplemental Table 1, differences in relative survival after 3, 5, and 10 y of follow-up are stated.

Clinically relevant and statistically significant (*P* < 0.05) survival differences prevail in PTC with larger tumor size and with lymph node involvement (intermediate risk). For a larger tumor size (pT3 or minimal extrathyroidal extension), a clinically relevant survival benefit of 1.3% in the RAI group begins to emerge after a follow-up time of approximately 8 y. With lymph node involvement, a higher survival rate in the RAI group appears after a few months (<12 mo) of follow-up, followed by a constant survival difference of approximately 2%. At very-long-term follow-up of approximately 150 mo, error bars begin to overlap at a stable survival difference of 1.5% (*P* = 0.359), whereas the number of patients followed up markedly reduces down to 211 patients at 200 mo. In high-risk PTC, a stable and robust relative survival benefit of more than 10% in the RAI group prevails after approximately 3 y.

In the very-low-risk and low-risk subgroups (including pT1bN0M0–pT2N0M0), no survival difference can be detected (the supplemental materials give details).

The survival difference in aggressive variants of PTC compared with classical PTC appears less clear for intermediate-risk patients (with lower patient number and higher uncertainty), qualitatively similar to the results of Holoubek et al. (16). The reason may be an a priori lower iodine uptake in more aggressive malignancies.

### FTC

**FIGURE 2.**
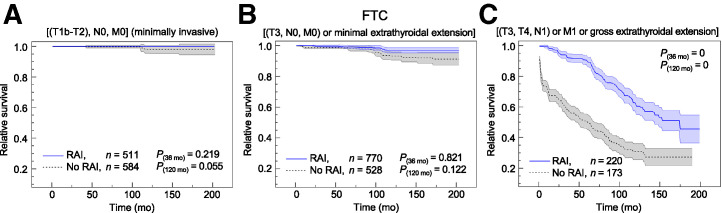
Relative survival including SE band in patients with FTC with or without RAI therapy based on SEER cancer database for low-risk minimally invasive (A), intermediate-risk (B), and high-risk (C) subgroups.

In Figure 2, relative survival rates with and without RAI are depicted for low-risk minimally invasive, intermediate-risk, and high-risk FTC. In the supplemental materials, relative survival of very-low-risk and low-risk FTC and relative survival of intermediate-risk minimally invasive FTC are depicted (Supplemental Figs. 3 and 4). In cases of minimally invasive FTC only in low-risk and intermediate-risk patients (T3N0M0 or minimal extrathyroidal extension), a sufficiently large number of patients were identified (*n* > 50) to meaningfully compare subgroups. In both cases, a trend toward higher survival rates in the RAI group than in the non-RAI group prevails. Even in the low-risk subgroup, a clear trend toward a higher relative survival rate of 2.0% is observed (*P* = 0.055). Qualitatively similar to T3 intermediate-risk PTC, the clinically relevant survival difference begins to emerge after approximately 100 mo of follow-up time.

In FTC, the relative survival rate tends to be higher in certain low-risk, intermediate-risk, and high-risk subgroups after RAI therapy, with more pronounced differences in relative survival in FTC than in PTC (Fig. 2; Supplemental Fig. 5). However, because of an overall lower number of patients, significance (*P* < 0.05) was reached only in the high-risk category after 10 y of follow-up. In particular, for the T1N1–T3N1 subgroup, no clinically meaningful conclusions can be drawn due to the low number of patients (Table 1). However, for the high-risk FTC subgroup, a relative survival benefit of 30.9% (*P* < 0.05) in the RAI group was observed. This is a larger difference than for the PTC subgroup of the same risk category. Without RAI, relative survival in high-risk FTC is reduced to an absolute value of approximately 27% after 11 y, pointing to the partially detrimental outcome of DTC in certain histologic subtypes and cancer stages without appropriate treatment. Clinically relevant survival differences for high-risk FTC already emerge after a few months.

Relative survival is not negatively affected in any DTC subgroup after RAI therapy compared with the non-RAI subgroups.

## DISCUSSION

This study analyzed SEER data from 2000 to 2020 comprising 101,087 patients with DTC, which were categorized into predefined risk categories for tumor recurrence. It showed a clear trend for a higher long-term (10-y) relative survival rate even in minimally invasive low-risk FTC after RAI therapy compared with the non-RAI group. For patients harboring PTC, a higher long-term survival rate with RAI therapy was demonstrated in cases with lymph node involvement. It was even more pronounced with distant metastasis, irrespective of T stage, emerging after less than 12 mo of follow-up time. In the subgroup with lymph node involvement, at a very-long-term follow-up of more than 150 mo, the difference in survival loses statistical significance. A noticeably reduced numbers of patients followed is the most likely reason.

For intermediate-risk T3 patients, a higher survival rate in the RAI group is shown emerging after a longer follow-up time of about 100 mo.

For small tumors without lymph node involvement (including pT1bN0M0–pT2N0M0), 10-y survival shows no difference with and without RAI therapy. To reasonably ensure that no lymph node involvement prevails, 6, 9, and 18 nodes need to be examined in cases of T1b, T2, and T3, respectively (22). Moreover, RAI not only serves as a treatment option but also may be an important stating tool, possibly first detecting lymph node metastases.

Only a single prospective randomized trial evaluated the effect of RAI therapy in low-risk DTC patients (ESTIMABL2). However, follow-up time was short (3 y), and FTC patients were underrepresented (18,19). Other prospective randomized studies—HiLo (23) and ESTIMABL1 (24)—focused on successful remnant ablation and did not compare survival with or without RAI therapy.

SEER is the largest database containing PTC and FTC patients with knowledge of TNM status, allowing long-term comparison of patients with and without RAI therapy. In the absence of long-term prospective randomized trials, retrospective analysis of SEER cohorts is at present the most important data source the assess the effect of adjuvant RAI.

Within the period studied (2000–2020), use of RAI has been drastically reduced in low- and intermediate-risk patients, according to the 2016 American Thyroid Association guideline (1). Therefore, a potential mismatch in the relation of patients with and without RAI therapy in this SEER review may prevail. However, as shown in Table 1 and Supplemental Table 1, the relation between the subgroups at 10-y follow-up remains stable, enabling a valid comparison in many subgroups. Results are clinically irrelevant only for some subgroups (e.g., FTC with T1N1–T3N1), particularly when few patients remain after applying the initial selection criteria (details are given in the Materials and Methods section).

Recently reported SEER database studies showed a trend toward better overall survival after RAI therapy even for low-risk patients (15). Yet inconsistencies with regard to cancer-specific survival exist (12–15). Overall survival is affected by any cause of death. Cancer-specific survival relies on the cause-of-death information provided. Therefore, it is subject to errors, mostly when misclassification of cause of death occurs.

Here, for the first time, to our knowledge, relative survival has been evaluated as a net survival measure in a database study on DTC. Relative survival relies on expected survival tables and therefore is inherently not affected by misclassification of cause of death; thus, it is considered more appropriate for rarer cancer types and older patients (17,21). In addition, for the first time, to our knowledge, patients were a priori stratified into risk categories, allowing independent direct comparison between risk categories for each histology subtype.

Although most database analysis focused on PTC patients, we also analyzed the rarer group of FTC patients. The benefit of RAI is usually not questioned in patients with widely invasive FTC; however, in less aggressive FTC, it may be questioned. In our study, even for low-risk minimally invasive FTC, a clear trend toward a higher relative survival rate of 2.0% at 10-y follow-up in the RAI group was observed (*P* < 0.1). The survival difference begins to emerge after long-term follow-up of approximately 100 mo, qualitatively similar to the period for PTC patients with the intermediate-risk T3 tumor stage.

Because survival for most DTC subgroups is comparably high, side effects of the treatment and age of the patients play important roles. If lymph node involvement remains undetected after not undergoing RAI therapy, surgery for relapse may become necessary, with a greater risk of side effects such as laryngeal nerve palsy. However, risk of side effects of RAI, such as xerostomia, particularly increase after high-dose or recurrent RAI therapy.

Our database research has shortcomings. The exact dosage of RAI applied, number of subsequent RAI treatments, and details with regard to surgical technique and number of additional surgeries cannot be clarified. In addition, the biochemical status (thyroglobulin) and the social status of the individuals cannot be specified, all of which may influence clinical outcome. The retrospective nature is an apparent shortcoming as well.

In addition, most risk stratification systems acknowledge the number and size of lymph nodes involved. Few (*n* < 5) and small (infiltration depth, <0.2 cm) lymph nodes are considered less aggressive. SEER allows differentiation between N0 and N1, but not precise number and infiltration depth. Consequently, we may have grouped patients in the intermediate-risk group despite being classified as low or very low risk per the guideline. In turn, this study had no patients with verified lymph node involvement in the low-risk category, which makes overestimation of the beneficial effect of RAI in low-risk patients unlikely.

## CONCLUSION

We present a retrospective cohort analysis of patients from the SEER database harboring PTC and FTC. Patients were a priori stratified into recurrence risk subgroups based on current guidelines (3). Long-term relative survival, with its strength as a robust measure of net survival, was compared for subgroups undergoing RAI therapy and not undergoing RAI therapy.

Depending on histologic subtype, the 10-y relative survival rate is higher in patients with low-, intermediate-, and high-risk DTC after undergoing RAI therapy: for FTC, a clear trend toward higher survival rates was observed, even for low-risk minimally invasive FTC. For PTC, a survival benefit prevails in the presence of lymph node involvement and larger tumor size and is most pronounced with distant metastasis. Benefits in relative survival of up to 30.9% (*P* < 0.05) were observed in the high-risk category.

Relative survival is not negatively affected in any subgroup undergoing RAI therapy compared with the subgroups not undergoing RAI therapy.

## DISCLOSURE

Alexander Drzezga discloses research support from Siemens Healthineers, Life Molecular Imaging, GE HealthCare, AVID Radiopharmaceuticals, Sofie, Eisai, Novartis/AAA, and Ariceum Therapeutics and has been a speaker or member of honorary or advisory boards for Siemens Healthineers, Sanofi, GE HealthCare, Biogen, Novo Nordisk, Invicro, Novartis/AAA, Bayer Vital, and Lilly. Alexander Drzezga holds stock in Siemens Healthineers, Lantheus Holding, Structured Therapeutics, and Lilly and a patent for ^18^F-JK-PSMA-7 (European patent EP3765097A1, January 20, 2021). Matthias Schmidt is a member of the Thyroid Committee and Guideline Committee of the German Society of Nuclear Medicine. No other potential conflict of interest relevant to this article was reported.

## Notes

KEY POINTS **QUESTION:** Does RAI therapy improve long-term relative survival in patients with DTC stratified according to recurrence risk categories? **PERTINENT FINDINGS:** Even in low-risk subgroups, depending on histologic subtype, a higher relative survival rate during long-term follow-up can be observed after undergoing RAI therapy. **IMPLICATIONS FOR PATIENT CARE:** RAI therapy may improve survival in selected patients even in low- to intermediate-risk subgroups and needs to be considered in the overall management of patients harboring DTC.
